# Analysis of Copper-Binding Proteins in Rice Radicles Exposed to Excess Copper and Hydrogen Peroxide Stress

**DOI:** 10.3389/fpls.2016.01216

**Published:** 2016-08-17

**Authors:** Hongxiao Zhang, Yan Xia, Chen Chen, Kai Zhuang, Yufeng Song, Zhenguo Shen

**Affiliations:** ^1^College of Agriculture, Henan University of Science and TechnologyLuoyang, China; ^2^College of Life Sciences, Nanjing Agricultural UniversityNanjing, China

**Keywords:** Cu stress, Cu-binding protein, H_2_O_2_ stress, immobilized metal affinity chromatography, germinating rice seed

## Abstract

Copper (Cu) is an essential micronutrient for plants, but excess Cu can inactivate and disturb the protein function due to unavoidable binding to proteins at the cellular level. As a redox-active metal, Cu toxicity is mediated by the formation of reactive oxygen species (ROS). Cu-binding structural motifs may alleviate Cu-induced damage by decreasing free Cu^2+^ activity in cytoplasm or scavenging ROS. The identification of Cu-binding proteins involved in the response of plants to Cu or ROS toxicity may increase our understanding the mechanisms of metal toxicity and tolerance in plants. This study investigated change of Cu-binding proteins in radicles of germinating rice seeds under excess Cu and oxidative stress using immobilized Cu^2+^ affinity chromatography, two-dimensional electrophoresis, and mass spectra analysis. Quantitative image analysis revealed that 26 protein spots showed more than a 1.5-fold difference in abundances under Cu or H_2_O_2_ treatment compared to the control. The identified Cu-binding proteins were involved in anti-oxidative defense, stress response and detoxification, protein synthesis, protein modification, and metabolism regulation. The present results revealed that 17 out of 24 identified Cu-binding proteins have a similar response to low concentration Cu (20 μM Cu) and H_2_O_2_ stress, and 5 out of 24 were increased under low and high concentration Cu (100 μM Cu) but unaffected under H_2_O_2_ stress, which hint Cu ions can regulate Cu-binding proteins accumulation by H_2_O_2_ or no H_2_O_2_ pathway to cope with excess Cu in cell. The change pattern of these Cu-binding proteins and their function analysis warrant to further study the roles of Cu ions in these Cu-binding proteins of plant cells.

## Introduction

Copper (Cu), an essential micronutrient required for growth and development in all plants, is a structural and catalytic cofactor of several proteins and enzymes involved in electron transfer and redox reactions. More than 100 proteins comprising two groups are estimated to have the ability to complex with Cu in *Arabidopsis*: Cu-binding proteins/chaperones and enzymes (Häensch and Mendel, 2009). However, excess Cu is toxic to most plants, causing a wide range of deleterious effects such as the inhibition of photosynthesis and pigment synthesis, damage to plasma membranes, and other metabolic disturbances. At the cellular level, excess Cu can inactivate and disturb the protein structure via unavoidable protein binding (Yruela, 2009). To control metal homeostasis and redox status, plants have several mechanisms of metal tolerance, including exclusion, compartmentalization, and binding to organic ligands such as organic acids, amino acids, peptides, and proteins (Hall, 2002; Yruela, 2009). Recently, the molecular and physiological basis for plant interactions with metals has attracted considerable interest. The identification of metal-binding proteins involved in the responses of plants to metal toxicity may improve our understanding regarding the mechanisms of metal toxicity and tolerance in plants.

Moreover, as a redox-active metal, Cu^+^ can catalyze the formation of reactive oxygen species (ROS) such as the superoxide anion (O${}_{2}^{•-}$), hydrogen peroxide (H_2_O_2_), and hydroxyl radical (HO·) via Fenton-type reactions (Schützendübel and Polle, 2002). ROS can oxidize proteins, unsaturated fatty acids, and nucleic acids, resulting in cellular damage and cell death. To scavenge ROS and alleviate their deleterious effects, plants have evolved various protective mechanisms that use superoxide dismutase (SOD), catalase (CAT), peroxidase (POD), ascorbate peroxidase (APX), and glutathione reductase (GR). Some antioxidant enzymes such as SOD have high affinities for binding to Cu, zinc (Groppa et al., 2008), manganese (Weeks et al., 2006), or iron (Fe). However, ROS can serve as signaling molecules for the induction of plant responses to environmental stresses such as metals (Babu et al., 2003; Maksymiec, 2007; Tamás et al., 2010). Cho and Seo (2005) reported that a reduced H_2_O_2_ accumulation increases cadmium (Cd)-tolerance in *Arabidopsis* seedlings. Exogenous H_2_O_2_ supplied to rice seedlings increased glutathione (GSH) levels and protected against subsequent Cd stress (Chao et al., 2009). H_2_O_2_ may be involved in the regulation of Cd- and heat shock-increased APX and GR activities in rice leaves (Chou et al., 2012). The improved Cd tolerance in rice seedlings by H_2_O_2_ may be due to stimulation of the antioxidant system and Cd sequestration (Hu et al., 2009). Although numerous physiological and biochemical analyses have examined the responses of plants to metal toxicity and the role of H_2_O_2_ as signaling molecules regulating metal-responsive protein accumulation in plants, the process remains unclear.

Immobilized metal affinity chromatography (IMAC) combined with mass spectrometry (MS) has been used to investigate the metal-binding proteome (She et al., 2003; Smith et al., 2004; Kung et al., 2006; Tan et al., 2010; Sun et al., 2011). This technique can separate proteins from biological samples based on specific interactions between proteins in solution and metal ions immobilized on a solid support (Porath et al., 1975; Sun et al., 2005). Metal ions are typically complexed with chelating ligands such as iminodiacetic acid (IDA). The proteins are separated according to their affinity for the chelated metal ions. In the bacterium *Streptococcus pneumoniae*, 232 and 166 putative metal-binding protein species were respectively isolated using a Cu- and Zn-IMAC column (Sun et al., 2011). Metals often bind proteins at specific coordination sites involving Cys, His, and Met residues (Harding, 2004). Smith et al. (2004) used a Cu-IMAC approach to enrich Cu-binding proteins in hepatocellular cells and reported nine putative metal-binding domains, namely, H–(X)_n_–H (*n* = 0–5) and C–(X)_m_–C (*m* = 2–4). Kung et al. (2006) identified 35 putative Cu-binding proteins in *Arabidopsis* roots, and found that 29 protein species possessed one or more of the H–(X)_n_–H (*n* = 0–5) and C–(X)_m_–C (*m* = 2–4) metal-binding motifs proposed by Smith et al. (2004). Kung et al. (2006) further identified the top six candidate motifs (H–(X)_5_–H, H–(X)_7_–H, H–(X)_12_–H, H–(X)_6_–M, M–(X)_7_–H, and H–(X)_3_–C), which accounted for 31 of the 35 proteins (89%). Tan et al. (2010) identified 35 weak and 48 strong Cu^2+^–IMAC-interactions in *Arabidopsis* mitochondria. Based on their data, 72% of the identified Cu-binding proteins contained one or more of the top six Cu-binding motifs (H–(X)_5_–H, C–(X)_7_–H, H–X–C, H–(X)_2_–M, M–(X)_3_–H, or M–(X)_7_-H). However, limited information is available on the metal-binding proteome in plants and other organisms under excess metal stress conditions.

Rice (*Oryza sativa* L.), an important food crop worldwide, is often used as a model for monocotyledons because of its well-established database. Several proteomic studies have been conducted on seed germination, growth regulation, and stress responses in rice (Ahsan et al., 2007; Aina et al., 2007; Yang et al., 2007; Zang and Komatsu, 2007; Zhang et al., 2009; Lee et al., 2010; Wu et al., 2011; Song et al., 2013). In a previous study, we developed a novel IMAC method, in which the IDA-Sepharose column was applied prior to a Cu-IMAC column to remove metal ions from protein samples for separating and isolating Cu-binding proteins from Cu-treated rice roots (Song et al., 2014). By comparing the difference of Cu-binding proteins in the roots of Cu-tolerant and Cu-sensitive rice varieties exposed to excess Cu (Chen et al., 2015), we had found some Cu-binding proteins involved in Cu tolerance in rice, but we did not know by which pathway these proteins were accumulated. We hypothesized that ROS signal molecules, especially those induced by Cu, might be involved in the Cu-binding proteins accumulation. In this study, we further identified soluble proteins isolated from the Cu-IMAC column that are regulated by Cu or H_2_O_2_. The aim of this report was to characterize the mechanisms involved in excess Cu stress responses and the role of H_2_O_2_ as a signaling molecule or redox substrate in the expression of soluble Cu-binding proteins in plants.

## Materials and methods

### Plant growth and treatment

Rice seeds (*O. sativa* L. cv. Wuyunjing No. 7, obtained from company of Nanjing Shenzhou Seed) were surface-sterilized with 5% (v/v) sodium hypochlorite (NaClO) for 15 min and thoroughly washed in distilled water. Each treatment was performed in triplicate. For one replicate, 100 seeds were randomly placed on moist filter paper in 200 mm Petri dish. The seeds were germinated in the dark at 25°C with renewal of distilled water every day. After 4 days, these germinating rice seeds were transferred to the mesh over 2.5 L vessel containing different concentrations of Cu sulfate pentahydrate (CuSO_4_·5H_2_O) solution (0–200μM) for 0–48 h, 1 mM ascorbic acid (Asc) for 12 h or 10 mM H_2_O_2_ solution for 6 h. A certain number of radicles from each replicate were obtained for the below experiments.

### Histochemical detection of H_2_O_2_

H_2_O_2_ formation *in situ* in rice radicles was visually detected based on the infiltration of 3,3′-diaminobenzidine (DAB) as described by Romero-Puertas et al. (2004) with minor modifications. Briefly, six radicles from each replicate (each Petri dish) were immersed in a 1 mg/mL solution of DAB (pH 3.8) and incubated at room temperature for 20 min in the absence of light. After staining, images were captured with a Coolpix 4500 digital camera (Nikon, Tokyo, Japan).

### H_2_O_2_ determination in extracts

The concentration of H_2_O_2_ in rice radicles from Cu-treated plants was measured by monitoring the A415 of the titanium-peroxide complex following the method described by Jiang and Zhang (2001). Absorbance values were calibrated to a standard curve established with 0.1–1.0 μM H_2_O_2_.

### Protein extraction

Rice seeds germinated for 4 days were treated with 10 mM H_2_O_2_ for 6 h or with 20 and 100 μM Cu for 12 h, referred as low and high concentration Cu treatment, respectively. Seeds germinated in deionized water without Cu and H_2_O_2_ were used as controls. Radicles were harvested and ground with a mortar and pestle in liquid nitrogen to obtain a fine powder, and then suspended in four volumes of protein binding buffer (20 mM sodium phosphate, pH 5.8, 500 mM NaCl, 0.1% (w/v) Triton X-100) containing 1 mM phenylmethyl sulfonyl fluoride (PMSF), incubated for 30 min at 4°C, and centrifuged for 30 min at 15,000 g at 4°C. The proteins in the supernatant were used for protein analysis, and the protein concentration was determined according to the Bradford method using bovine serum albumin (0, 0.2, 0.4, 0.6, 0.8, 1.0 mg/mL) as the standard (Bradford, 1976).

### Separation and isolation of Cu-binding proteins based on Cu-IMAC

Experimental design for proteomic analysis of Cu-binding proteins in rice radicles was shown in Supplementary Figure S1. The used method for separating and isolating Cu-binding proteins was based on the Cu-IMAC method of Song et al. (2014). Protein samples were pre-chromatographed on a column with IDA-Sepharose for removing metal ions from proteins samples before flowing over a Cu-IDA-Sepharose column (referred to as Cu-IMAC) for separating Cu-binding proteins. IDA-Sepharose and Cu-IMAC were connected in tandem with a tube (inner diameter of 0.5 mm). For the pre-chromatography column, IDA-Sepharose with a 2 mL bed volume for each column was poured into a 10-mL glass column with an inner diameter of 10 mm and washed with 10 mL of water at a rate of 0.5 mL/min. For the Cu-IMAC column, IDA-Sepharose with a 2 mL bed volume for each column was poured and washed with 10 mL of water at a rate of 0.5 mL/min, after which the bed volume of 0.2 M CuSO_4_ was applied to the column, followed by washing with 15 mL distilled water to remove excess Cu ions at a rate of 0.5 mL/min. Columns were equilibrated with 10 bed volumes of binding buffer at a linear flow rate of 0.5 mL/min after they were connected in tandem.

A total of 20 mg of protein sample solution was loaded onto the pre-chromatography column at a linear flow rate of 0.25 mL/min, after which the column was washed with binding buffer until the ultraviolet absorbance of the effluent from the Cu-IMAC column reached baseline. Binding buffer containing 10 mM imidazole was added to the per-chromatography column at a linear flow rate of 0.5 mL/min until the UV-absorbance of the effluent from the Cu-IMAC column reached baseline, suggesting that non-specific proteins were removed. The Cu-binding proteins were eluted with elution buffer (10 mM sodium acetate, 500 mM NaCl, pH 5.5) containing 40 mM imidazole at a linear flow rate of 0.5 mL/min until the UV-absorbance of the effluent from the Cu-IMAC column reached baseline.

The Cu-binding proteins eluted from Cu-IMAC were precipitated with four volumes of cooled 10% (w/v) acetone (containing 0.07% (w/v) dithiothreitol, DTT) overnight at −20°C for 1 h, followed by centrifugation for 15 min at 10,000 g at 4°C. Afterward, the pellets were dissolved in lysis buffer (7 M urea, 2 M thiourea, 4% (w/v) CHAPS, 0.2% (w/v) Bio-lytes pH 3–10, 65 mM DTT). Protein concentrations were assayed using a Bio-Rad RC DC Protein Assay Kit 1.

### Two-dimensional electrophoresis (2-DE), gel scanning, and image analysis

For each replicate, 100 μg of total protein extract was loaded onto IPG dry strips (17 cm, pH 4–7 linear gradient; Bio-Rad, Hercules, CA, USA) during the rehydration step (13 h), followed by focusing for a total of 60,000 V·h using a Protean IEF Cell (Bio-Rad). Following isoelectric focusing (IEF), the gel strips were equilibrated for 15 min in 5 mL equilibration buffer containing 0.375 M Tris–HCl (pH 8.8), 6 M urea, 20% (v/v) glycerol, 2% (w/v) sodium dodecyl sulfate (SDS), and 2% (w/v) DTT. The strips were then equilibrated in the same buffer as described above, but including 2.5% w/v iodacetamide instead of DTT. SDS-polyacrylamide gel electrophoresis (PAGE) in the second dimension was performed using 12% SDS-polyacrylamide gels sealed with 0.5% agarose. Electrophoresis was performed at 50 V for the first 30 min, followed by 150 V for 8 h using a Protean Plus Dodeca Cell apparatus (Bio-Rad). Protein spots were visualized using MS-compatible silver staining (Yan et al., 2000). To prevent the gels being overexposed, the developing course was divided into two steps: firstly, the developing solution was drained off after becoming yellow; secondly, the developing course was terminated when the small protein spots begin to become clear.

The gels were scanned using the image scanner UMAX Powerlook III (UMAX Technologies, Dallas, TX, USA) at 300 dpi resolution; image and data analyses of the gels were performed using PDQuest software (version 8.0; Bio-Rad) and a multivariate statistical package (DeCyder EDA, Unscrambler, Samespots), which can automatically deal with missing values during analysis (Valledor and Jorrín, 2011). The abundance of spot mean a summation of the pixel intensities localized within the defined spot area, which obtained by PDQuest (Bio-Rad) image analysis software. Spot quantity was normalized in the “total quantity of valid spot” mode for possible staining differences between gels. Duplicate 2-DE gels were run for each treatment from three independent tissue extractions, only spots with significant and reproducible changes were considered to represent differentially accumulated proteins. The results for the control and Cu- or H_2_O_2_-treated samples were analyzed for differences using Student's *t*-test with a significance level of 5%. Protein spots were selected for MS analysis when a difference of 1.5-fold or greater was observed in the level of accumulation between the treatment and control.

### In-gel digestion of protein, MS analysis, and functional classification

Protein spots were excised and destained (Gharahdaghi et al., 1999). The samples were incubated in 50 mM ammonium bicarbonate for 5 min, dehydrated with acetonitrile (ACN), and dried. The peptides were extracted with 60% ACN and 0.1% trifluoroacetic acid after the proteins were digested with trypsin, and were then extracted and desalted with ZipTip C18 columns (Millipore, Bedford, MA, USA). The peptide solution was saturated with α-cyano-4-hydroxycinnamic acid and then air-dried on an MS sample plate.

Peptide mass spectra were obtained using a 4700 Proteomics Analyzer MALDI-TOF/TOF™ mass spectrometer (Applied Biosystems, Framingham, MA, USA) in positive ion reflector mode. The subsequent MS/MS analysis was performed in a data-dependent manner, and the 10 most abundant ions fulfilling certain preset criteria were subjected to high-energy collisional dissociation (CID) analysis. The collision energy was set to 1 keV, and nitrogen was used as the collision gas.

All protein spectra were submitted for database searching using the online MASCOT program (http://www.matrixscience.com) against NCBInr databases (http://www.ncbi.nlm.nih.gov/protein). The taxonomic category selected was *Oryza sativa*. The searching parameters were as follows: 0.15 Da mass tolerance for peptides and 0.25 Da mass tolerance for TOF–TOF fragments, one allowed trypsin miscleavage, Cys carbamidomethylation as a fixed modification, and Met oxidation as a variable modification. Only significant hits, as defined by the MASCOT probability analysis (*P* < 0.05), were accepted.

Kyoto Encyclopedia of Genes and Genomes (KEGG) (http://www.genome.jp/kegg/ or http://www.kegg.jp/) was used to predict molecular function, biological processes, and significant pathways involved in response to stress.

### Total RNA isolation, cDNA synthesis, and quantitative RT-PCR

Total RNA was extracted using the RNA simple Total RNA Kit (LifeFeng, Shanghai, China) according to the manufacturer's instructions and then converted to cDNA after DNase I treatment using a PrimeScript™ RT Master Mix (TaKaRa). Real-time quantitative RT-PCR was performed on a MyiQ Real-Time PCR Detection System (Bio-Rad Hercules, CA, USA) using SYBR Premix Ex Taq (TaKaRa). The primers for protein mRNA were listed in Supplementary Table S1. The PCR protocol included an initial 7-min incubation at 95°C for complete denaturation followed by 40 cycles at 94°C for 30 s, 60°C for 30 s, and 72°C for 30 s. The specificity of the PCR amplification was examined based on a heat dissociation curve (65–95°C) following the final cycle. Normalized relative expression was calculated using the 2^−ΔΔCt^ (cycle threshold) method.

### Statistical analysis

Data were analyzed using SPSS ver. 16.0 (Statistical Package for Social Science for Windows, SPSS, Inc., Chicago, IL, USA). All values reported in this paper are means ± SE (*n* = 3) of three separate experiments. Means denoted by the same letter did not significantly differ at *P* < 0.05 according to Duncan's multiple range test.

## Results

### Induction of Cu on H_2_O_2_ production in radicles

**Figure 1:**
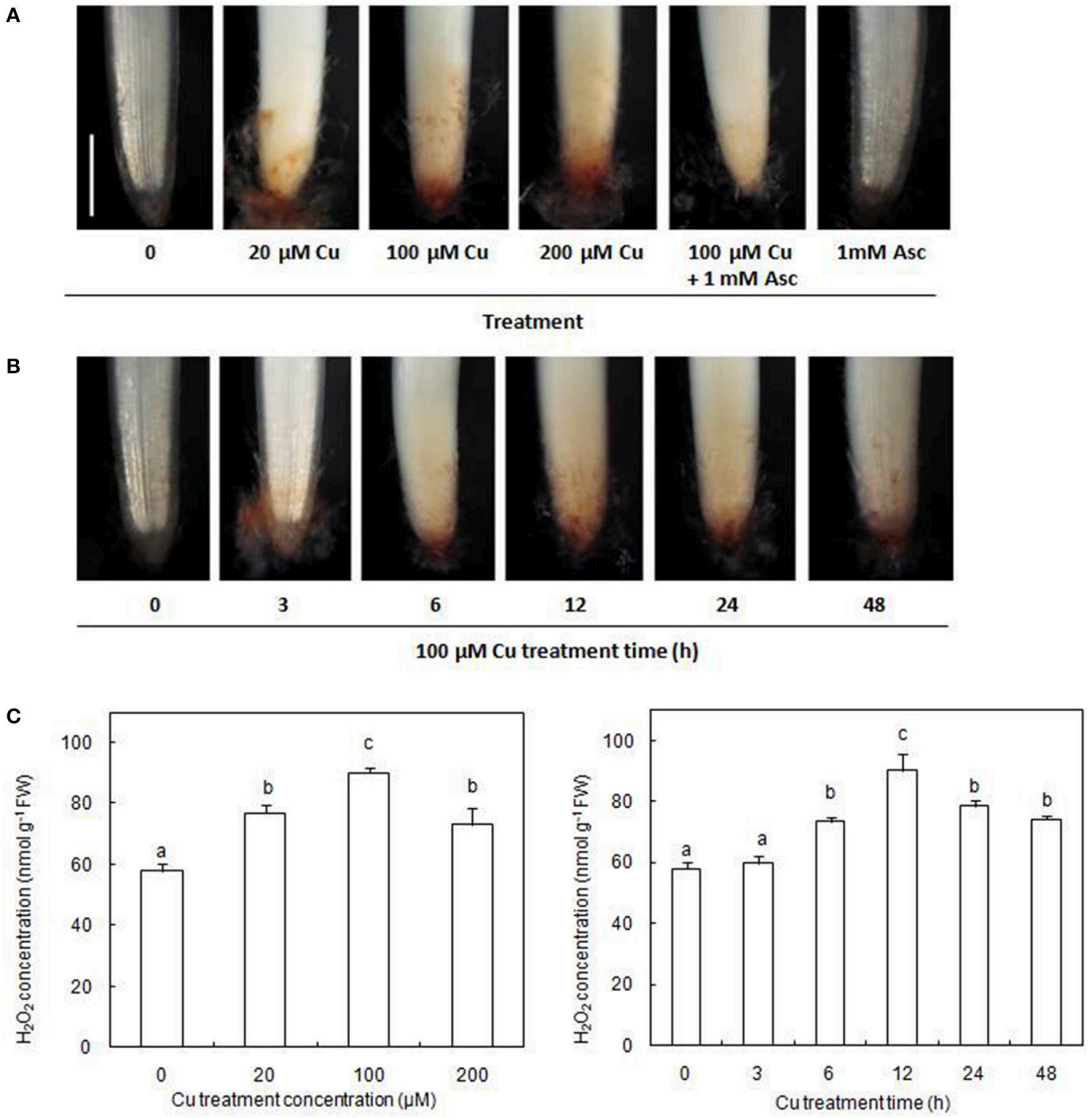
**Cu-induced changes in H_2_O_2_ accumulation on rice radicles. (A)** Histochemical detection of H_2_O_2_ in rice radicles under different treatments. **(B)** Histochemical detection of H_2_O_2_ in rice radicles under varied Cu treatment times. **(C)** The concentration of H_2_O_2_ in rice radicles under different Cu treatments. Germinating rice seeds were treated with CuSO_4_ (0, 20, 100, and 200 μM) solution and 1 mM ascorbic acid (Asc) solution for 12 h, or treated with 100 μM CuSO_4_ solution for 0, 3, 6, 12, 24, and 48 h. Subsequently radicles from Cu-treated plants were incubated in 1 mg/mL solution of 3,3-diaminobenzidine (DAB, pH 3.8) for 20 min, or were homogenized and the H_2_O_2_ content assayed by spectrophotometry. Bar, 1 mm.

The accumulation of H_2_O_2_ in the radicles of germinating rice seeds was examined using histochemical DAB staining. Our results showed that exposure to excess Cu for 12 h caused an evident accumulation of H_2_O_2_ in the radicles (Figure 1A). The production of Cu-induced H_2_O_2_ could be decreased by infiltration with the H_2_O_2_ scavenger, Asc. In the presence of 100 μM Cu, H_2_O_2_ accumulation in radicles gradually increased during the first 12 h of exposure and then decreased slightly but remained higher than the control (Figure 1B). The concentrations of H_2_O_2_ assayed by spectrophotometry were consistent with the results of histochemical detection by DAB staining (Figure 1C).

### Identification of Cu-binding proteins modulated by Cu and H_2_O_2_

**Figure 2:**
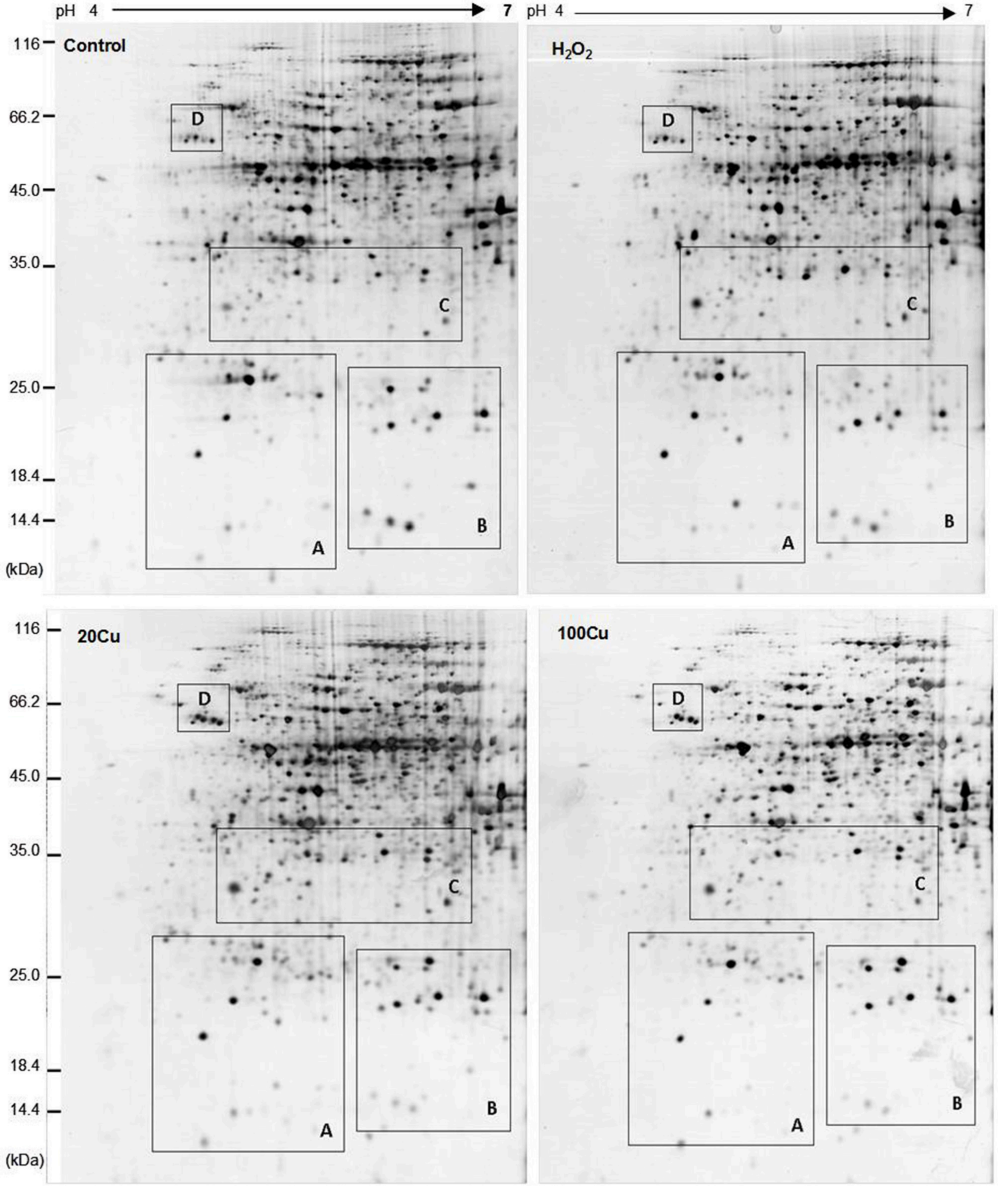
**Representative 2-DE maps of copper-binding proteins obtained from radicles of germinating rice seeds treated with Cu and H_2_O_2_ via Cu-IMAC plus IDA-Sepharose pre-chromatography**. Germinating rice seeds were treated with control (deionized water without Cu and H_2_O_2_), 10 mM H_2_O_2_ for 6 h, 20 and 100 μM Cu for 12 h. A 20 mg proteins extracts from radicles of germinating rice seeds was loaded onto the column with IDA-sepharose to removal metal ions in protein samples before onto Cu-IMAC. These Cu-binding proteins eluted from a Cu-IMAC column were subjected to 2-DE separation. One-hundred microgram of total protein were loaded onto IPG dry strips (17 cm, pH 4–7 linear gradient), the second dimension was carried out using 12% SDS-PAGE. The protein spots were visualized by mass spectrometry compatible silver staining.

**Figure 3:**
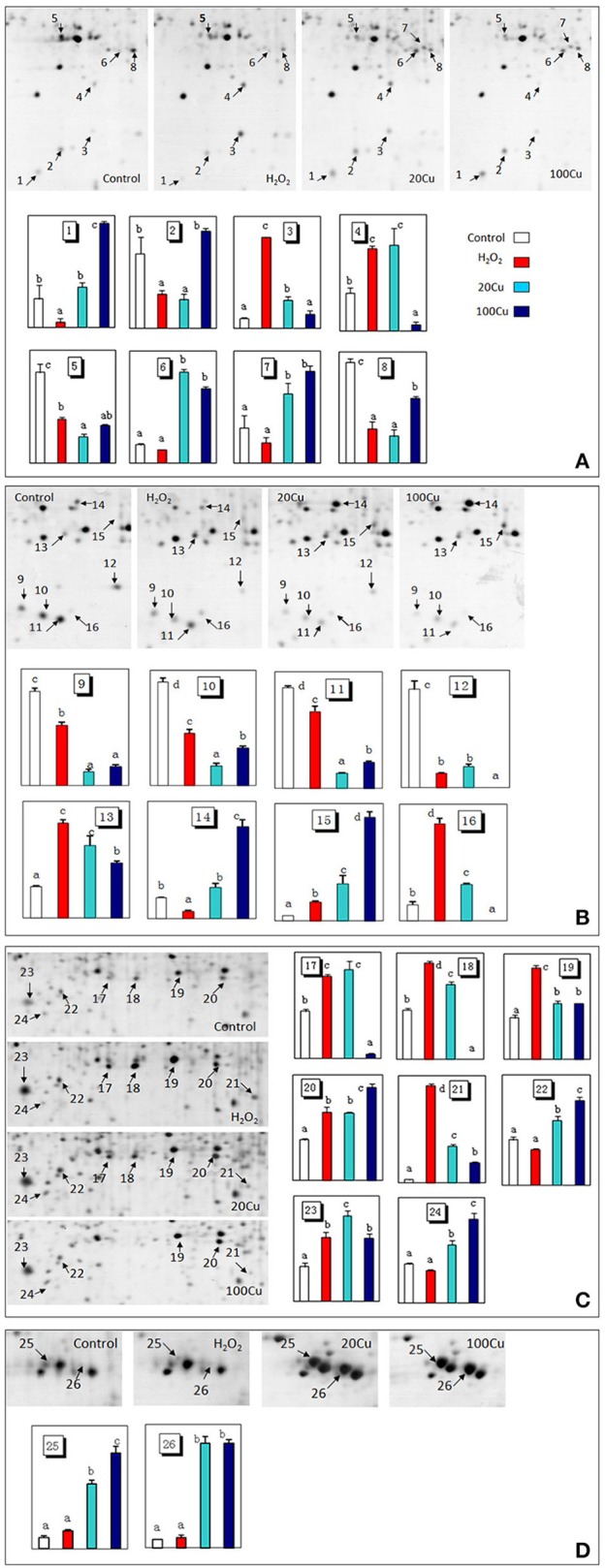
**Enlargements of the framed areas (A), (B), (C), and (D) shown in Figure 2 and the relative abundance of differentially accumulated proteins**. Arrows indicate the differentially expressed proteins in response to Cu and H_2_O_2_ stress. The vertical axis (abundance) mean a summation of the pixel intensities localized within the defined spot area, which obtained by PDQuest (Bio-Rad) image analysis software.

UV detections to Cu-binding proteins of rice radicles via Cu-IMAC were shown in Supplementary Figure S2. The Cu-binding protein yields from the control, H_2_O_2_-treated, 20 μM Cu, and 100 μM Cu-treated rice radicles, estimated with the percents of peak area (of total peak area), were not significantly different. Protein maps produced from 2-DE gels showed a high reproducibility among the three independent extractions (Figure 2, Supplementary Figure S3). When analyzed using PDQuest, 780 ± 15, 772 ± 25, 793 ± 13, and 695 ± 24 proteic spots were identified in the range of pH 4–7 and relative molecular masses of 10–120 kDa with the control, H_2_O_2_-treated, 20 μM Cu and 100 μM Cu-treated rice radicles, respectively. The significantly differential spot patterns between 100 μM Cu treatment and the other treatments might be explained by differing degrees of protein loss resulting from the Cu-binding proteins eluted from the Cu-IMAC column. Among all of the spots, 656 spots were present with all four treatment, other protein spots were treatment-specific. 26 protein spots, exhibited more than 1.5-fold differences in the abundances under at least one treatment (20, 100 μM Cu, or 10 mM H_2_O_2_) compared to the control. In order to observe more clearly, these protein spots were artificially divided into four regions (A, B, C and D) in gels, and the four regions were enlarged and shown in Figure 3.

Compared with the control, the H_2_O_2_ treatment increased the abundances of 11 spots and decreased that of nine spots among the 26 protein spots. Among the 11 H_2_O_2_-increased protein spots, six protein spots were simultaneously increased under both Cu treatments (20 and 100 μM Cu), and five protein spots were increased under 20 μM Cu. Among nine protein spots decreased under H_2_O_2_, six protein spots were similarly decreased under both Cu treatment, one spot decreased under 20 μM Cu treatment. In addition, the abundances of six protein spots only increased under Cu treatment and unaffected under H_2_O_2_ treatment (Supplementary Figure S4).

The Cu-binding proteins in 26 spots were analyzed using MALDI-TOF/TOF MS, and all spectra of proteins were submitted to a NCBInr protein database search using the online MASCOT program. Supplementary Table S2 shows the identity of Cu-binding proteins in 26 spot after a database search. The molecular masses (Mr) and isoelectric point (pI) values of each identified protein are listed in Table 1. These identified Cu-binding proteins were found to be involved in different cellular responses and metabolic processes, including antioxidative defense (6 proteins), stress response and detoxification (4 proteins), protein synthesis (5 proteins), protein modification (1 protein in 2 spots), protein metabolism (2 protein in 3 spots), carbohydrate metabolism (3 proteins), nucleotide metabolism (1 protein), and secondary metabolite metabolism (2 proteins).

**Table 1:** **Differentially accumulated Cu-binding proteins of rice radicles identified by MS/MS**.

**Spot no.**	**NCBI Accession no.**	**Protein name**	**Average-fold change^a^**			**Exp Mr/pI**	**Theo Mr/pI**	**PM^b^**	**SC^c^ %**	**Score**
			**H_2_O_2_**	**20Cu**	**100Cu**					
**INVOLVED IN ANTIOXIDATIVE DEFENSE**										
6	ADM86864	Glutathione S-transferase 2	–	5.1↑	4.1↑	24.7/5.50	24.3/5.77	3(2)	14	106
8	AAP13093	L-ascorbate peroxidase	3.0↓	3.8↓	1.6↓	24.9/5.72	27.2/5.31	2(1)	11	66
13	BAD03019	Putative quinone- oxidoreductase QR2	3.0↑	2.3↑	1.7↑	23.0/6.27	21.6/6.08	2(2)	15	120
14	AAC64007	Glutathione S-transferase GSTF2	2.9↓	–	4.5↑	26.1/6.28	24.3/5.77	5(4)	27	307
15	AAQ01200	Peroxiredoxin	4.5↑	8.3↑	23.3↑	23.8/6.51	24.2/5.97	7(5)	35	309
16	AAA33917	Copper/zinc superoxide dismutase	5.3↑	2.3↑	↓	17.7/6.27	15.3/5.71	2(2)	15	175
**INVOLVED IN STRESS RESPONSE AND DETOXIFICATION**										
1	AAF85972	Pathogenesis-related protein PR-10a	5.9↓	–	3.7↑	14.2/4.81	16.9/4.95	4(4)	34	235
2	ABA99548	Pathogenesis-related protein Bet v I family protein	2.2↓	2.6↓	–	16.7/5.02	17.2/4.96	4(3)	36	150
17	AAM12483	Cytochrome P450-like protein	1.7↑	1.9↑	10.3↓	36.0/5.63	58.1/6.28	3(2)	10	79
23	AAB23484	Salt stress-induced protein	1.8↑	2.5↑	1.8↑	33.1/5.01	15.2/5.00	6(6)	66	656
**INVOLVED IN PROTEIN SYNTHESIS**										
3	BAD54334	Susceptibility homeodomain transcription factor	9.4↑	2.9↑	–	18.2/5.31	18.4/5.30	3(3)	26	263
9	ABA98689	Putative eukaryotic translation initiation factor 5A-2	1.6↓	6.8↓	5.0↓	17.6/5.91	17.8/5.6	5(5)	45	364
10	ABF98987	Putative eukaryotic translation initiation factor 5A-2	2.0↓	5.3↓	2.8↓	17.0/6.02	17.9/5.87	6(4)	40	270
11	AAC67555	Translation initiation factor 5A	1.3↓	6.8↓	3.9↓	16.8/6.18	17.7/5.77	6(6)	35	479
21	ABL74569	Elongation factor 2	30.0↑	11.4↑	6.1↑	32.0/6.52	95.0/5.85	5(1)	4	173
**INVOLVED IN PROTEIN MODIFICATION**										
25	AAX85991	Protein disulfide isomerase	–	5.8↑	8.6↑	62.2/4.89	57.0/4.95	8(5)	16	355
26	AAX85991	Protein disulfide isomerase	–	12.9↑	12.8↑	63.3/4.80	57.0/4.95	8(7)	18	466
**INVOLVED IN PROTEIN METABOLISM**										
18	AAU44086	Putative legumin	2.0↑	1.5↑	↓	35.9/5.80	38.5/5.81	5(3)	15	233
19	AAU44086	Putative legumin	2.2↑	1.3↑	1.3↑	36.7/6.08	38.5/5.81	6(5)	15	345
24	AAX11351	Cathepsin B-like cysteine protease	–	1.5↑	2.2↑	31.7/5.22	40.4/6.25	8(8)	23	550
**INVOLVED IN CARBOHYDRATE METABOLISM**										
7	AAB63603	Triosephosphate isomerase	–	2.0↑	2.6↑	25.1/5.58	27.8/6.60	3(1)	14	81
12	ABR25593	Glyceraldehyde-3-phosphate dehydrogenase	7.0↓	4.7↓	↓	19.0/6.53	28.5/8.62	7(4)	20	302
20	BAD07953	Putative NADPH-dependent mannose 6-phosphate reductase	1.7↑	1.7↑	2.3↑	35.9/6.28	37.5/5.88	6(6)	21	382
**INVOLVED IN NUCLEOTIDE METABOLISM**										
22	BAB89118	Cytidine/deoxycytidine deaminase-like	–	1.5↑	2.2↑	32.9/5.20	32.2/5.13	6(4)	22	295
**INVOLVED IN SECONDARY METABOLISM**										
4	ACN65507	Arginine decarboxylase 2	2.2↑	2.3↑	6.3↓	21.8/5.32	67.7/6.45	5(5)	11	382
5	AAM13448	Chalcone-flavonone isomerase	2.1↓	3.5↓	2.4↓	25.9/5.20	23.9/5.15	4(4)	31	398

a *Spot abundance is accumulated as the ratio of intensities of proteins between stress and control. Fold changes indicate a statistically significant difference (P < 0.05) between treated samples and control samples by Duncan's test; ↑, up-regulated; ↓, down-regulated (alone↓, disappearance of spot); –, no change. H_2_O_2_, 20 Cu, and 100 Cu represent 100 μ M H_2_O_2_, 20 μ M Cu and 100 μ M Cu treatment concentrations, respectively*.

b PM, number of peptides matched.

c SC, sequence coverage by MS/MS.

### Analyses of metal-binding motifs

In this study, among 24 proteins in 26 spots identified, 18 proteins contained one or more of nine metal-binding motifs reported by Smith et al. (2004), and 20 protein species contained one or more of the top six motifs (H–(X)_5_–H, H–(X)_7_–H, H–(X)_12_–H, H–(X)_6_–M, M–(X)_7_–H, and H–(X)_3_–C) reported by Kung et al. (2006) in *Arabidopsis* roots (Table 2). Fifteen proteins contained motifs reported by Smith et al. (2004) and the top six motifs by Kung et al. (2006). However, one protein (spots 18 and 19; putative legumin) contained neither the motifs reported by Smith et al. (2004) nor the top six motifs reported by Kung et al. (2006).

**Table 2:** **Potential Cu-binding motifs of identified proteins**.

**Spot no.**	**Protein name**	**Reported motif I^a^**	**Reported motif II^b^**
1	Pathogenesis-related protein PR-10a	–	MX_7_H
2	Pathogenesis-related protein Bet v I family protein		MX_7_H
3	Susceptibility homeodomain transcription factor	HX_2_H	MX_7_H
4	Arginine decarboxylase 2	HH; HXH; HX_3_H; HX_5_H	HX_5_H; HX_7_H; HX_12_H; HX_3_C; MX_7_H
5	Chalcone-flavonone isomerase	HXH	–
6	Glutathione S-transferase 2	–	HX_4_H; CX_3_C
7	Triosephosphate isomerase	HH; HXH; HX_2_H; HX_3_H; HX_4_H	HX_4_H
8	L-ascorbate peroxidase	HX_5_H	HX_5_H; MX_7_H
9	Putative eukaryotic translation initiation factor 5A-2	HH; HXH; HX_3_H; HX_5_H	HX_5_H
10	Putative eukaryotic translation initiation factor 5A-2	HH; HXH; HX_3_H; HX_5_H	HX_5_H
11	Translation initiation factor 5A	HH; HXH; HX_3_H; HX_5_H	HX_5_H
12	Glyceraldehyde-3-phosphate dehydrogenase	HH; HX_4_H; HX_5_H; CX_3_C	HX_5_H
13	Putative quinone- oxidoreductase QR2	HH	–
14	Glutathione S-transferase GSTF2		HX_4_H; CX_3_C
15	Peroxiredoxin	HX_5_H	HX_5_H; HX_12_H
16	Copper/zinc superoxide dismutase	HXH; HX_2_H; HX_4_H	HX_7_H
17	Cytochrome P450-like protein	HX_2_H; HX_3_H; HX_4_H	HX_7_H; HX_6_M
18,19	Putative legumin	–	–
20	Putative NADPH-dependent mannose 6-phosphate reductase	HXH; CX_4_C	–
21	Elongation factor 2	CX_4_C	MX_7_H
22	Cytidine/deoxycytidine deaminase-like	HX_5_H	HX_5_H; HX_7_H; HX_3_C
23	Salt stress-induced protein	–	HX_7_H
24	Cathepsin B-like cysteine protease	CX_2_C; CX_4_C	HX_7_H; HX_12_H; HX_3_C
25,26	Protein disulfide isomerase	HX_5_H; CX_2_C	HX_5_H

a *Motifs that were reported by Smith et al. (2004)*.

b *Motifs that were reported by Sookoian et al. (2008). “–”indicates not present; H, C, and M indicates respectively the three amino acids His, Cys and Met, X indicates any one of 20 amino acids*.

### Transcriptional analysis of genes for some Cu-binding proteins

**Figure 4:**
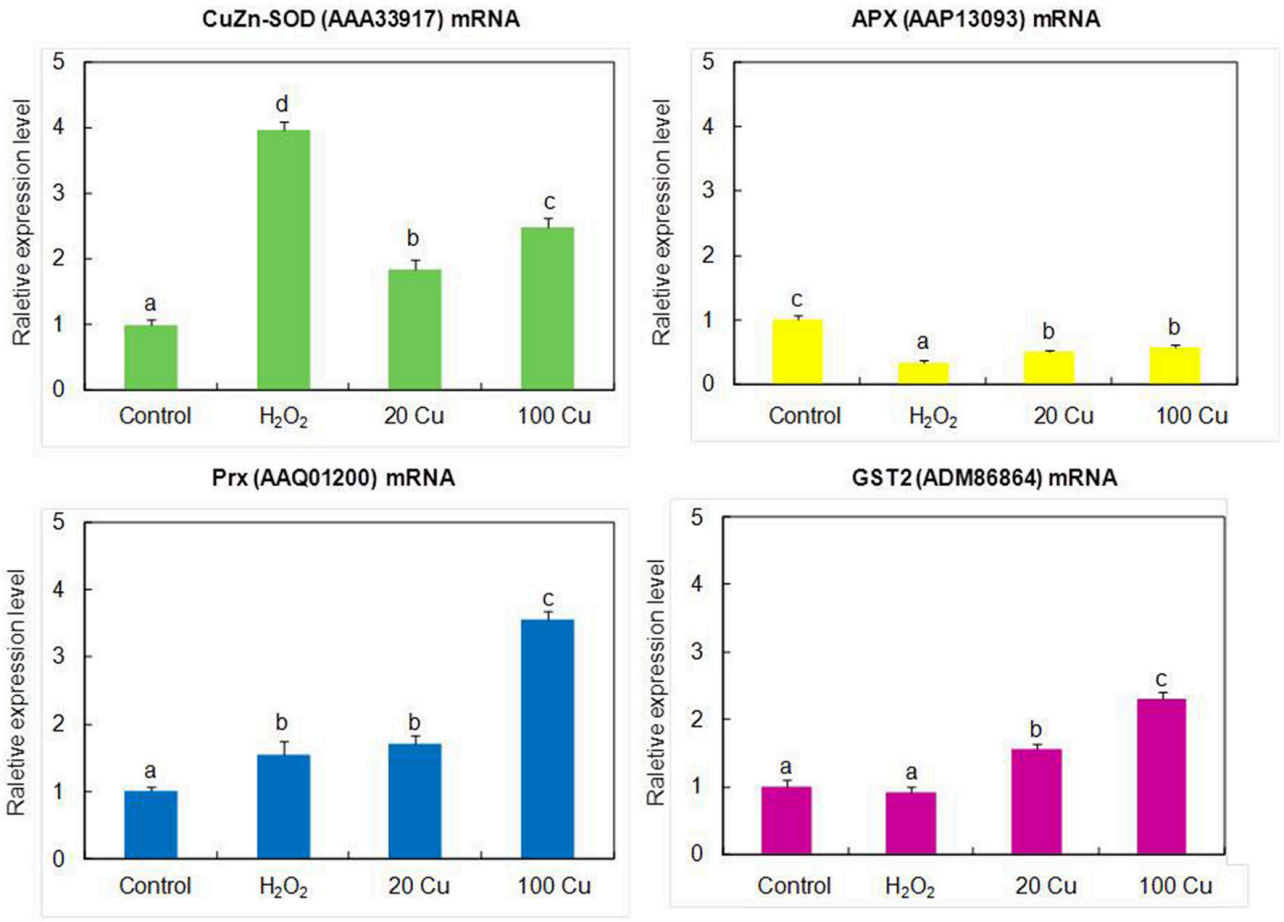
**Real-time quantitative RT-PCR analyses the gene expression of the identified proteins**. The mRNA levels of CuZn-SOD (AAA33917), APX (AAP13093), Prx (AAQ01200), GST2 (ADM86864) were analyzed by real-time quantitative RT-PCR. The germinating seeds treated with 0, 20, and 100 μM Cu for 12 h or with 10 mM H_2_O_2_ for 6 h.

In order to assess the correlation between mRNA expression and protein accumulation, Real-time quantitative RT-PCR was applied to four mRNAs of identified Cu-binding proteins, copper/zinc superoxide dismutase (CuZn-SOD, spot 16), L-ascorbate peroxidase (APX, spot 8), peroxiredoxin (Prx, spot 15), and glutathione S-transferase 2 (GST2, spot 6), involved in antioxidative defense (Figure 4). By compared with the corresponding spots of Figure 3, the expression analyses of four genes were consistent with the proteins accumulation except for CuZn-SOD mRNA change under high Cu treatment, indicate that the accumulation of these proteins have been largely regulated at the transcriptional level.

## Discussion

### Cu-induced accumulation of H_2_O_2_ and Cu-binding proteins

Numerous studies have shown that excess Cu can induce the formation of ROS (including H_2_O_2_) and cause oxidative stress. In this study, the formation of H_2_O_2_ was observed with increasing Cu concentrations and with Cu treatment time in the Cu-treated radicles of rice (Figure 1). Accumulation of H_2_O_2_ has also been observed in other Cu-exposed plant species using histochemical staining (Tewari et al., 2006; Sgherri et al., 2007; Zhang et al., 2008, 2010). Because H_2_O_2_ is relatively stable and diffusible through membranes, it is known to modulate gene expression and participate in various physiological processes (Neill et al., 2002; Ahmad et al., 2008). So far, no proteins simultaneously response to H_2_O_2_ and Cu stress were reported by searching web of science.

In the present study, 24 Cu-IMAC-binding proteins in 26 spots were identified that were differentially accumulated at least by one treatment (20, 100 μM Cu, or 10 mM H_2_O_2_). The same protein (e.g., protein disulfide isomerase and putative legumin) in varied spots is possible since the spot change its position in the gel due to changes in pI or Mr as a consequence of post-translational modifications. Among these identified proteins, elongation factor EF-2 (EF-2), GST, Prx, APX, quinone-oxidoreductase QR2 (QR2), protein disulfide isomerase (PDI), glyceraldehyde 3-phosphate dehydrogenase (GAPDH), triosephosphate isomerase (TPI), NADPH-dependent mannose 6-phosphate reductase (M6PR), and cytidine/deoxycytidine deaminase-like (CDC) have been identified as Cu-IMAC-binding proteins in *Arabidopsis* (Kung et al., 2006; Tan et al., 2010), soybean (Wang et al., 2014), microalgae (Smith et al., 2014), and rice (Song et al., 2014). Moreover, the analogs of eukaryotic translation initiation factor 5A (eIF5A), transcription factor (TF), and SOD, such as translation initiation factor Tu, G, 3A, and 4A, iron-dependent transcriptional regulator, and Fe-SOD were identified as Cu-IMAC-binding proteins in hepatoma cells (Smith et al., 2004), in *Arabidopsis* (Kung et al., 2006) and *S. pneumoniae* (Sun et al., 2011). SODs are metalloenzymes found in three different molecular forms containing Cu and Zn (CuZn-SOD), Mn (Mn-SOD), or Fe (Fe-SOD) as prosthetic metals. However, to the best of our knowledge, the other eight proteins, including pathogenesis-related protein (PR) known as PR-10a, and Bet v I family protein (PR-b), cytochrome P450-like protein (CYP-L), salt stress-induced protein (SSI), legumin, cathepsin B-like cysteine protease (CBCP), arginine decarboxylase (ADC), and chalcone-flavonone isomerase (CHI), have not been reported as Cu-IMAC-binding proteins in plants. The present data showed that the abundance of 11 spots increased under exogenous H_2_O_2_ treatment out of 17 spots increased under 20 μM Cu, and that all 20 μM Cu-decreased spots decreased under H_2_O_2_ (Table 1, Supplementary Figure S3), which may be ascribed to the same amount H_2_O_2_ produced by low concentration Cu as H_2_O_2_ treatment. However, H_2_O_2_ or Cu at high levels can cause oxidative stress and cell damage, which could be the reason that the abundance of five protein spots are decreased under 100 μM Cu stress but increase under H_2_O_2_ and 20 μM Cu stress.

### Cu-binding proteins simultaneously accumulated under low Cu and H_2_O_2_ treatments

Four identified Cu-binding proteins such as CuZn-SOD (spot 16), QR2 (spot 13), Prx (spot 15), and APX (spot 8), displayed similar behavior under 20 μM Cu and H_2_O_2_ treatments, which may play important roles in plant antioxidant defense responses. SODs are key players in the antioxidant defense system through the dismutation of O${}_{2}^{•-}$ to H_2_O_2_. SODs as metal chelators may also regulate the intracellular Cu level. In plants, quinones are redox-active compounds that oxidize the thiol groups of proteins and GSH. QR2 catalyzes two electron reductions of quinones to hydroquinones (Malakshah et al., 2007; Vannini et al., 2012). Prx, which consists of many different thiol-disulfide exchange proteins, such as thioredoxins and glutaredoxins, is an H_2_O_2_-scavenging enzyme that reduces H_2_O_2_ to H_2_O, and Prx possesses a highly reactive Cys that is oxidized to form a disulfide bond coupled with the reduction in H_2_O_2_ (Dietz et al., 2006). Thus, increase of these Cu-binding proteins may alleviate Cu-induced damage by decreasing free Cu^2+^ activity in the cytoplasm and/or scavenging ROS.

Notably, the abundance changes of specific Cu-binding proteins responded differentially to excess Cu: the abundances of Prxs and QR2 increased, while that of APX decreased. The abundance of CuZn-SOD increased under 20 μM Cu but decreased under higher levels of Cu (100 μM). These different changes of antioxidative enzymes following exposure to excess Cu may be due to their varied functions. Excess Cu increased SOD expression (Sunkar et al., 2006; Cohu et al., 2009; Zhang et al., 2010) and activities (Tewari et al., 2006; Zhang et al., 2008, 2010). However, Cu ions can be dangerous to cellular compartments as free ions. Thus, high Cu treatment (100 μM Cu) decreased the abundance of CuZn-SOD in this study. APX and CAT are two major scavengers of H_2_O_2_. APX is present throughout the cell and has a higher substrate affinity in the presence of Asc as a reductant. Cu and H_2_O_2_ have been reported to increase APX expression and activity (Lee et al., 2007). In this study, the abundance of APX decreased under excess Cu and exogenous H_2_O_2_ treatments. Decrease of APX could lead to the accumulation of H_2_O_2_ and enhance oxidative stress. Similar decrease of APX was observed in rice leaves (Wan and Liu, 2008) and tobacco cells (Vannini et al., 2012) in response to H_2_O_2_ stress at high doses (50 mM) or over extended times (6 h). H_2_O_2_ was suggested to directly inhibit APX activity by causing protein oxidation at concentrations over a threshold value (de Pinto et al., 2006). The abundance changes of GST (spots 6 and 14) responded differentially to excess Cu and exogenous H_2_O_2_ treatment. A major function of GSTs is to detoxify a variety of hydrophobic and electrophilic compounds by catalyzing their conjugation with GSH (Jwa et al., 2006). Consistent with our results, an increased GSTs was detected in Cu-treated (Song et al., 2013, 2014) and H_2_O_2_-treated rice (Wan and Liu, 2008). In contrast, a decrease in GST levels in rice exposed to Cu (Ahsan et al., 2007), H_2_O_2_ (Vannini et al., 2012), and selenium (Se) (Wang et al., 2012) has been observed.

The gene products of four identified antioxidant proteins (CuZn-SOD, APX, Prx and GST2) showed similar changes obtained from proteomics experiments except for CuZn-SOD change under high Cu treatments (Figure 4). Previous studies showed that Cu availability is the major factor that determines whether Fe-SOD or CuZn-SOD are expressed (Cohu et al., 2009), the CuZn-SOD accumulation is mediated by a microRNA, miR398, which targets CuZn-SOD mRNA for degradation under some condition (Sunkar et al., 2006), and CuZn-SOD proteins accumulated only when Cu ions were available for final assembly and stability. Thus, it is possible that the abundance decrease of CuZn-SOD protein spot under high Cu treatment was not consistent with the results of gene expression analyses.

Three proteins including PR-b, CYP-L, and SSI out of four Cu-binding proteins involved in the stress response and detoxification displayed similar behavior under low concentration Cu and H_2_O_2_ treatment. In plants, CYP proteins are involved in the synthesis of fatty acids, lignin, hormones, and flavonoids, as well as xenobiotic metabolism in higher plants (Schuler and Werck-Reichhart, 2003). In this study, the abundance of one CYP-L (spot 17) increased slightly under 20 μM Cu and H_2_O_2_ but significantly decreased under 100 μM Cu treatment. Li et al. (2009) observed an increase of the CYP-like protein in soybean at 2 h post inoculation. In contrast, decrease of CYP proteins was observed in Cu-treated rice germinating embryos (Zhang et al., 2009) and a Cd-treated *Phytolacca americana* leaf (Zhao et al., 2011). Wan and Liu (2008) observed that one putative salt-induced protein increased under H_2_O_2_ in rice leaves. In this study, Cu and H_2_O_2_ treatments significantly increased the abundance of SSI (spot 23), but its function in Cu-stressed plants remains unknown. PR proteins play a role in a wide range of cell functions, including cell wall rigidification, signal transduction, and antimicrobial activity (Markovic-Housley et al., 2003). Elevated levels of ROS have been reported to induce PR proteins in rice (Jwa et al., 2006). The increase of PR proteins has also been observed in Cu-treated *Phaseolus vulgaris* (Cuypers et al., 2005), *Elsholtzia splendens* (Li et al., 2009), and rice (Zhang et al., 2009). In this study, treatment with H_2_O_2_ and Cu decreased or did not affect the abundance of PR-10a (spot 1) and PR-b (spot 2), although 100 μM Cu increased that of PR-10a. The opposite change patterns of PR proteins by excess Cu suggest that they have different roles.

All Cu-binding proteins involved in protein synthesis displayed similar behavior under low concentration Cu and H_2_O_2_ treatments (Table 1). The abundances of susceptibility homeodomain transcription factor (SHTF, spot 3), EF-2, and (spot 21) increased under excess Cu and H_2_O_2_, excluding the high Cu treatment that did not affect that of SHTF and decreased that of legumin. In contrast, the abundances of eIF5A (spot 11) and eIF5A-2 (spots 9 and 10) decreased under both Cu and H_2_O_2_. eIF5A was also thought to play a role in translation elongation (Saini et al., 2009) and other aspects of RNA metabolism such as RNA export (Liu et al., 2008). The expression of eIF5A in plants usually increases in response to abiotic stress (Li et al., 2009; Xu et al., 2011; Meng et al., 2014; Parkash et al., 2014). In agreement with our result, a significant decrease of eIF5A was observed in rice after a long-term salt stress (Parker et al., 2006), which may be associated with premature senescence. EFs (EF1A, EF1B, and EF-2) are fundamental regulatory proteins of the translational elongation step in higher plants, as well as other eukaryotic organisms. EF-2 catalyzes GTP-dependent translocation of peptidyl-tRNA from the A site to the P site of the ribosome during peptide chain elongation (Browne and Proud, 2002). In this study, the abundances of EF-2 increased by 30.0-, 11.4-, and 6.1-fold in the presence of H_2_O_2_, low and high concentration Cu, respectively. Similar increase of EF-2 was observed in *Schizosaccharomyces pombe* in response to H_2_O_2_ stress (Weeks et al., 2006). In contrast, decrease of the EF-2 protein was observed in Cu-treated *E. splendens* roots (Li et al., 2009), Cd-treated *P. americana* (Zhao et al., 2011), and B-deficient *Brassica napus* (Wang et al., 2010).

Legumin is a major storage protein in plant seeds, including α and basic polypeptides of 40 and 20 kDa, respectively, bound by a disulfide bridge (Sabir et al., 1973). This protein contained neither the motifs reported by Smith et al. (2004) nor the top six motifs reported by Kung et al. (2006), but contained 8 of 117 potential metal-binding motifs (C-(X)n-C, C-(X)n-H, C-(X)n-M, H-(X)n-C, H-(X)n-H, H-(X)n-M, M-(X)n-C, M-(X)n-H, and M-(X)n-M, where *n* = 0–12) reported by Kung et al. (2006). Cu caused a reduction in the germination rate of bean, which increased the level of storage proteins compared to the control (Karmous et al., 2011). In this study, the abundances of legumin (spots 18 and 19) increased under excess Cu and H_2_O_2_. It is unknown whether increase of legumin protein abundance may alleviate Cu-induced damage by decreasing free Cu^2+^ activity in the cytoplasm or be a consequence of Cu toxicity and oxidative stress.

Two enzymes (GAPDH and M6PR) involved in carbohydrate metabolism and two enzymes (ADC and CHI) involved in secondary metabolism displayed similar behavior under Cu and H_2_O_2_ treatments. Treatments with both Cu and H_2_O_2_ decreased the abundance of GAPDH (spot 12) and increased M6PR (spot 20). *Arabidopsis* Cytosolic GAPDH may be a potential target of H_2_O_2_-dependent oxidation in plant protein extractions (Hancock et al., 2005). TPI and GAPDH are important enzymes in the glycolytic pathway. Here, the decrease of GAPDH and increase of TPI may favor the accumulation of glyceraldehyde 3-phosphate under stress conditions. M6PR, a key enzyme in mannitol biosynthesis, catalyzes the conversion of mannose 6-phosphate into mannitol 1-phosphate. Overexpression of M6PR genes from celery and *Arabidopsis* result in increased tolerance to salt stress (Sickler et al., 2007; Chan et al., 2011). Treatments with Cu and H_2_O_2_ increased abundance of ADC (spot 4) and decreased that of CHI (spot 5). Cu-induced increases in ADC activity were also observed in previous reports (Groppa et al., 2008; Xu et al., 2011). The roles of ADC and CHI as Cu-binding proteins in the tolerance to Cu and oxidative stresses are still unknown.

### Cu-binding proteins accumulated under Cu not H_2_O_2_ treatment

Whereas five identified Cu-binding proteins including GST (spot 6), PDI (spots 25 and 26), CBCP (spot 24), TPI (spot 7), and CDC (spot 22) were increased only under low and high Cu but unaffected under H_2_O_2_ stress, which hint Cu ions can regulate the Cu-binding proteins accumulation by no H_2_O_2_ pathway. PDI has been identified as a Cu-binding protein in previous reports (Smith et al., 2004; Song et al., 2014). PDI is a thioredoxin superfamily oxidoreductase from the endoplasmic reticulum, and catalyzes a wide range of thiol-disulfide exchange reactions, including oxidation, reduction, and isomerization, and also displays chaperone, calcium-binding, and Cu-binding activity (Hatahet and Ruddock, 2009; Laurindo et al., 2012). Overexpression of PDI gene from *Methanothermobacter thermoautotrophicum* enhances mercury tolerance in transgenic rice (Chen et al., 2012). Proteomic analyses showed that PDI accumulation increased in rice roots in the presence of Cu (Song et al., 2014), in rice leaves in the presence of H_2_O_2_ (Wan and Liu, 2008), and in soybean leaves during salt stress (Ma et al., 2012). Here, the abundance of PDIs was markedly higher in Cu-treated rice, which can enhance Cu tolerance in germinating rice seed by binding Cu and thiol-disulfide exchange reactions. In contrast, PDIs accumulation were down-regulated by Cu stress in roots of the tolerant plant *E. splendens* (Li et al., 2009), by H_2_O_2_ in rice root apoplasts (Zhou et al., 2011), and by flooding stress in soybean roots (Khatoon et al., 2012).

## Conclusions

**Figure 5:**
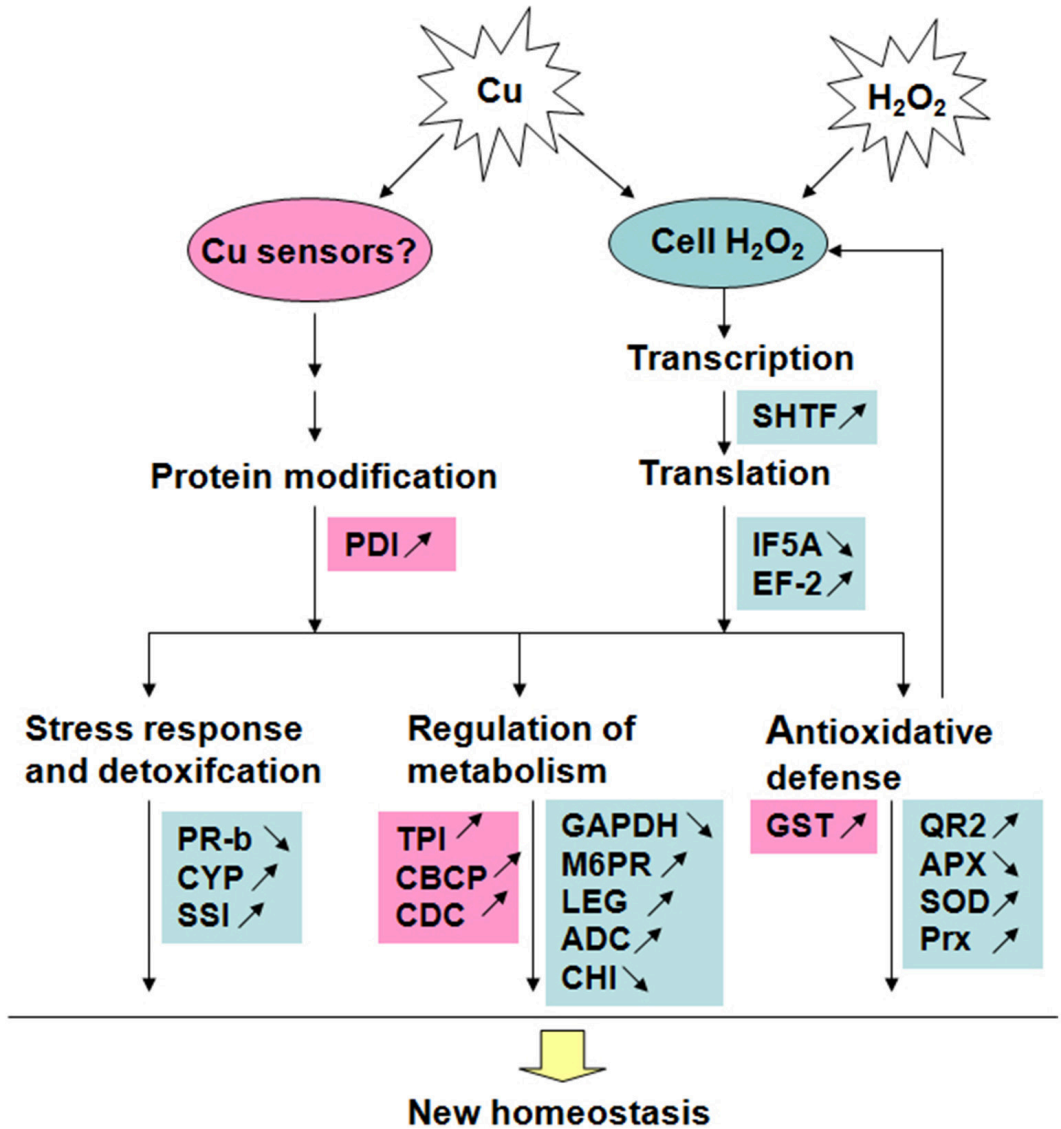
**A putative model of Cu-binding proteins in rice radicles to Cu and H_2_O_2_ stress responses**. Red blocks show proteins accumulated only under Cu treatment; Blue blocks show proteins accumulated under treatment with low concentration Cu and H_2_O_2_ in the same way. The proteins with accumulation increase are marked by “↑” and those with decrease marked by “↓”.

The present results revealed that 17 out of 24 identified Cu-binding proteins have a similar response to 20 μM Cu and H_2_O_2_ stress in rice radicles. These Cu-binding proteins involved in antioxidative defense, stress response, and detoxification, protein synthesis and metabolism, and can play important roles on reconstructing homeostasis of cell under stress condition by H_2_O_2_ signal pathway. The accumulation of five identified Cu-binding proteins were up-regulated by 20 and 100 μM Cu but unaffected by H_2_O_2_, which hint Cu ions can regulate Cu-binding proteins accumulation by no H_2_O_2_ pathway to cope with excess Cu in cell. A putative model of Cu-binding proteins in rice radicles to Cu and H_2_O_2_ stress responses was shown in Figure 5. Further studies are required to clarify the roles of Cu ions in these putative Cu-binding proteins in plant cells to determine if they are passive molecular targets of metal ions or active participants in metal tolerance.

## Author contributions

ZS and HZ designed research. HZ, YX, YS, and KZ conducted sampling, biochemical and data analysis. YS, CC, and KZ contributed with proteomic analysis. HZ, CC, and ZS wrote the manuscript. All authors read, reviewed and approved the manuscript.

### Conflict of interest statement

The authors declare that the research was conducted in the absence of any commercial or financial relationships that could be construed as a potential conflict of interest.
