# Supplementary material for: Analysis of Copper-Binding Proteins in Rice Radicles Exposed to Excess Copper and Hydrogen Peroxide Stress
Source: Front Plant Sci. 2016 Aug 17;7:1216. doi: 10.3389/fpls.2016.01216 (PMC4987373; doi:10.3389/fpls.2016.01216)
Supplement: Supplementary file 1 [file DataSheet1.zip › Data Sheet-zhang/FPLS OsCuBP H2O2_Supplementary_Material.dotx]

**Analysis of Copper-Binding Proteins in Rice Radicles Exposed to Excess Copper and Hydrogen Peroxide Stress**

**Hongxiao Zhang ^a^, Yan Xia ^b^, Chen Chen ^b^, Kai Zhuang ^b^ , Yufeng Song ^b^, Zhenguo Shen ^b ,*^**

^a^ College of Agriculture, Henan University of Science and Technology, Luoyang 471023, China

^b^ College of Life Sciences, Nanjing Agricultural University, Nanjing 210095, China

* Corresponding author: Zhenguo Shen

Email: [zgshen@njau.edu.cn](mailto:zgshen@njau.edu.cn)

# Supplementary Tables

# Supplementary Table S1∣The primers for protein mRNA.

| Protein  (NCBI no.) | Forward primer | Reverse primer |
| --- | --- | --- |
| CuZn-SOD (AAA33917) | TCATTGGCAGAGCCGTCGTTGT | AGTCCGATGATCCCGCAAGCAA |
| APX  (AAP13093) | CACCCAGGAAGGGAGGACAAACC | TGACCGCCAGAGAGGGCAACAAT |
| Prx  (AAQ01200) | TTCGTTTCTGCTCCCAGTGCTAA | AGTCGTGGATGCGGATCTTGC |
| GST2 (ADM86864) | GGCGACCTCAAGGAGTCAGCA | CATCATCGGACGGATTAGGCACT |
| OsActin | TTATGGTTGGGATGGGACA | AGCACGGCTTGAATAGCG |

**Supplementary Table S2∣Differentially accumulated proteins identified by MS/MS.**

| Spot no. | NCBI  accession no. | Protein name | PM *^a^* | Score | MS/MS peptide sequence＞95% C.I. (Indv. ion score) |
| --- | --- | --- | --- | --- | --- |
| 1 | AAF85972 | Pathogenesis-related protein PR-10a | 4(4) | 235 | LNPAAGVGSTYK(45); VAVCDAASHVLK(39);  MIEDYLVAHPAEYA(68); APACVSDEHAVAVSAER(83) |
| 2 | ABA99548 | Pathogenesis-related protein Bet v I family protein | 4(3) | 150 | IVVCDSATHVLK(36); SHSTETKLEATGDATCVAK(58); LTVEYELEDGASLSPEQEK(49) |
| 3 | [BAD54334](http://www.ebi.ac.uk/ena/data/view/BAD54334) | Susceptibility homeodomain transcription factor | 3(3) | 263 | SGAEVHTGHELCER(102); QTQAGGATHTFDTIGK(82); QVWYAGEVTAFVEQGR(79) |
| 4 | ACN65507 | Arginine decarboxylase 2 | 5(5) | 382 | VESAGGGGAFK(103); HNLFSGPTLVR(64); GLTTMPYLNDYKPPK(51);  VATENGVWPMVEPLMK(43); IVSVELGPTAEEVIGTMR(121) |
| 5 | AAM13448 | Chalcone-flavonone isomerase | 4(4) | 398 | DSSVPEGAVAAAAIENR(108); AAGVYTDAEGAAADKFK(109);  FTAIGVYLEEGAAVPALAK(65); ALCEAVLDSIIGEHGVSPAAK(116) |
| 6 | ADM86864 | Glutathione S-transferase 2 | 3(2) | 106 | GEHKAPDHLAR(46); NPFGQVPALQDGDLFLWESR(51) |
| 7 | AAB63603 | Triosephosphate isomerase | 3(1) | 81 | FFVGGNWKVIACVGETLEQR(76) |
| 8 | AAP13093 | L-ascorbate peroxidase | 2(1) | 66 | APPPEGRTPAELSHAANAGLDIAVR(48) |
| 9 | ABA98689 | Putative eukaryotic translation initiation factor 5A-2 | 5(5) | 364 | TYPQQAGTIR(59); LPTDDNLLSQIK(99); CHFVAIDIFTAK(102); DLVVTVMSAMGEEQICALK(38); KLEDIVPSSHNCDVPHVNR(68) |
| 10 | ABF98987 | Putative eukaryotic translation initiation factor 5A-2 | 6(4) | 270 | NGHIVIK(37); TYPQQAGTIR(62); CHFVAIDIFNGK(79); DDLRLPSDEALLTQIK(43); |
| 11 | AAC67555 | Translation initiation factor 5A | 6(6) | 479 | TYPQQAGTIR(57); LPTDDSLLGQIK(84); CHFVAIDIFNGK(92); DDLRLPTDDSLLGQIK(54); LEDIVPSSHNCDVPHVNR(94); KLEDIVPSSHNCDVPHVNR(100) |
| 12 | ABR25593 | Glyceraldehyde-3-phosphate dehydrogenase | 7(4) | 302 | KVVISAPSK(38);YDTVHGQWK(59); DAPMFVVGVNEK(79);  TLLFGEKEVTVFGCR(37) |
| 13 | BAD03019 | Putative quinone- oxidoreductase QR2 | 2(2) | 120 | GGSPYGSGTFAGDGSR(37); VATELELQQAFHQGK(83) |
| 14 | [AAC64007](http://www.ebi.ac.uk/ena/data/view/AAC64007) | Glutathione S-transferase GSTF2 | 5(4) | 307 | VVEENLEK(65); VLEVYEAR(72); GEHKAPDHLAR(37);  LYGSTLSWNVTR(82); NPFGQVPALQDGDLFLWESR(51) |

**Supplementary Table S2∣Continued**

| Spot no. | NCBI  accession no. | Protein name | PM *^a^* | Score | MS/MS peptide sequence＞95% C.I. (Indv. ion score) |
| --- | --- | --- | --- | --- | --- |
| 15 | AAQ01200 | Peroxiredoxin | 7(5) | 309 | ALHIVGPDKK(49); DIEAYKPGNR(47); LSFLYPACVGR(49);  FPQGFDTADLPSGK(66); HAVATPVNWKPGER(61) |
| 16 | AAA33917 | Copper/zinc superoxide dismutase | 2(2) | 175 | EHGAPEDETR(76); AVVVHADPDDLGK(99) |
| 17 | AAM12483 | Cytochrome P450-like protein | 3(2) | 79 | ALRDFSGDVFKRNAAK (36);  FVYKLIRDRSDELSNTKAHDTDSR(40) |
| 18 | AAU44086 | Putative legumin | 5(3) | 233 | AWDLAESDAVK(66); RIDSEIFFAPN(44); VLDTHVEGGNLFIVPR(89) |
| 19 | AAU44086 | Putative legumin | 6(5) | 345 | VAYVLQGK(40); LPEPSAADR(46); WDLAESDAVK(72);  RIDSEIFFAPN(52); VLDTHVEGGNLFIVPR(107) |
| 20 | BAD07953 | Putative NADPH-dependent mannose 6-phosphate reductase | 6(6) | 382 | TPAQLVLR(42); DLIHSALR(73); REDLFITTK(47);  SIGISNYDIFLTR(90); LWNSDHGHVVEACK(86);  IKPAVNQIETHPYFQR(44) |
| 21 | ABL74569 | Elongation factor 2 | 5(1) | 173 | EGALAEENMR(61) |
| 22 | BAB89118 | cytidine/deoxycytidine deaminase-like | 6(4) | 295 | VYAGVNLEFR(52); FPVGAVGLGASGR(60);  AVAVSHMPCGHCR(47); IIVTSDAEDGCAPEWR(67) |
| 23 | AAB23484 | Salt stress-induced protein | 6(6) | 656 | LLGVTIYSSDAIR(118); SGTLIDAIGIYVHP(108); KLLGVTIYSSDAIR(113); EFSIPLQDSGHVVGFFGR(118); EISGTHGPVYDLADIVTYLK(121);  SIAFNYIGVDGQEYAIGPWGGGEGTSTEIK(79) |
| 24 | AAX11351 | Cathepsin B-like cysteine protease | 8(8) | 550 | HFSVNAYR(66); GWGDDGYFK(62); KHFSVNAYR(61); HITGGMMGGHAVK(78); PGCEPAYPTPVCEK(90); GTNECGIEEDVVAGMPSTK(99);  GVVTDECDPYFDQVGCK(93) |
| 25 | AAX85991 | Protein disulfide isomerase | 8(5) | 355 | GDAAVERPLVR(40); EAEGIVEYLKK(53); VVVADNVHDFVFK(100);  SDYDFGHTLHANHLPR(36); VVTFDKNPDNHPYLLK(68) |
| 26 | AAX85991 | Protein disulfide isomerase | 8(7) | 466 | NIQEYKGPR(40); GDAAVERPLVR(37); TADEIVDFIKK(64);  VVVADNVHDFVFK(104); AHVEPDQIVSWLK(88);  SDYDFGHTLHANHLPR(50); VVTFDKNPDNHPYLLK(56) |

*^a^* PM, number of peptides matched.

# Supplementary Figure

**Supplementary Figure Captions**

**Supplementary Figure S1**∣**Experimental design for proteomic analysis of Cu-binding proteins in rice radicles.**

**Supplementary Figure S2**∣**UV detection to Cu-binding proteins of rice radicles via Cu-IMAC.Ⅰ**no specific Cu-binding proteins.**Ⅱ**specific Cu-binding proteins. Germinating rice seeds were treated with control (deionized water without Cu and H_2_O_2_), 10 mM H_2_O_2_ for 6 h, 20 µM and 100 µM Cu for 12 h. A 20 mg protein extracts from rice radicles was loaded onto the column with IDA-sepharose to removal metal ions in protein samples before onto Cu-IMAC.

**Supplementary Figure S3**∣**The specific Cu-binding proteins eluted from a Cu-IMAC column were subjected to 2-DE separation.** Images of all 2-DE gels made from the three biologically independent replicates (R1-3) of protein extracts from radicles of germinating rice seeds were treated with control (deionized water without Cu and H_2_O_2_), 10 mM H_2_O_2_ for 6 h, 20 µM and 100 µM Cu for 12 h. 100 μg of protein were loaded onto IPG dry strips (17 cm, pH 4–7 linear gradient), the second dimension was carried out using 12% SDS-PAGE and marker molecular masses of 14.4, 18.4, 25.0, 35.0, 45.0, 66.2 and 116 kDa.

**Supplementary Figure S4**∣**Venn diagram analysis of the differentially accumulated protein spots.** The numbers of protein spots with increased **(A)** or decreased **(B)** abundance under treatment of H_2_O_2_, 20 µM and 100 µM Cu are shown in the different segments.
