# Supplementary figures and images for: Analysis of Copper-Binding Proteins in Rice Radicles Exposed to Excess Copper and Hydrogen Peroxide Stress
Source: Front Plant Sci. 2016 Aug 17;7:1216. doi: 10.3389/fpls.2016.01216 (PMC4987373; doi:10.3389/fpls.2016.01216)

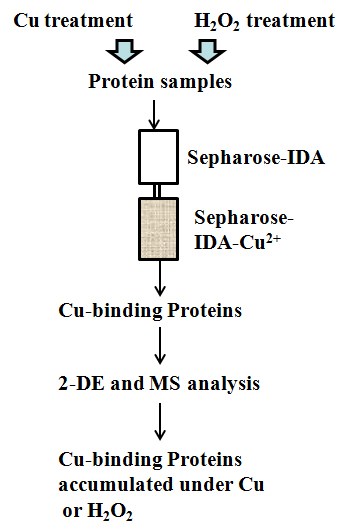

Supplement: Supplementary file 1 [file DataSheet1.zip › Data Sheet-zhang/Supplementary Figure S1.jpg]

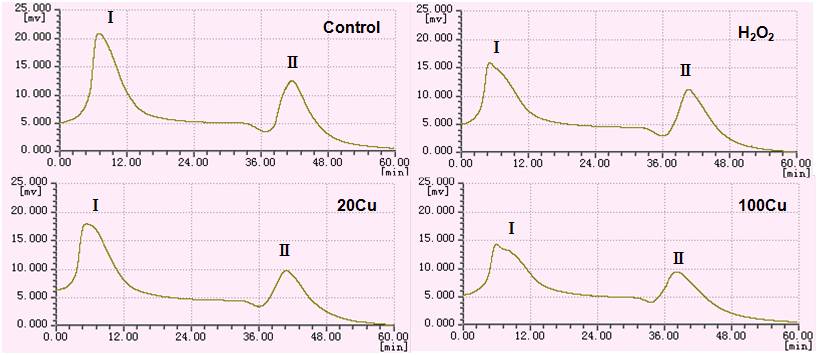

Supplement: Supplementary file 1 [file DataSheet1.zip › Data Sheet-zhang/Supplementary Figure S2.jpg]

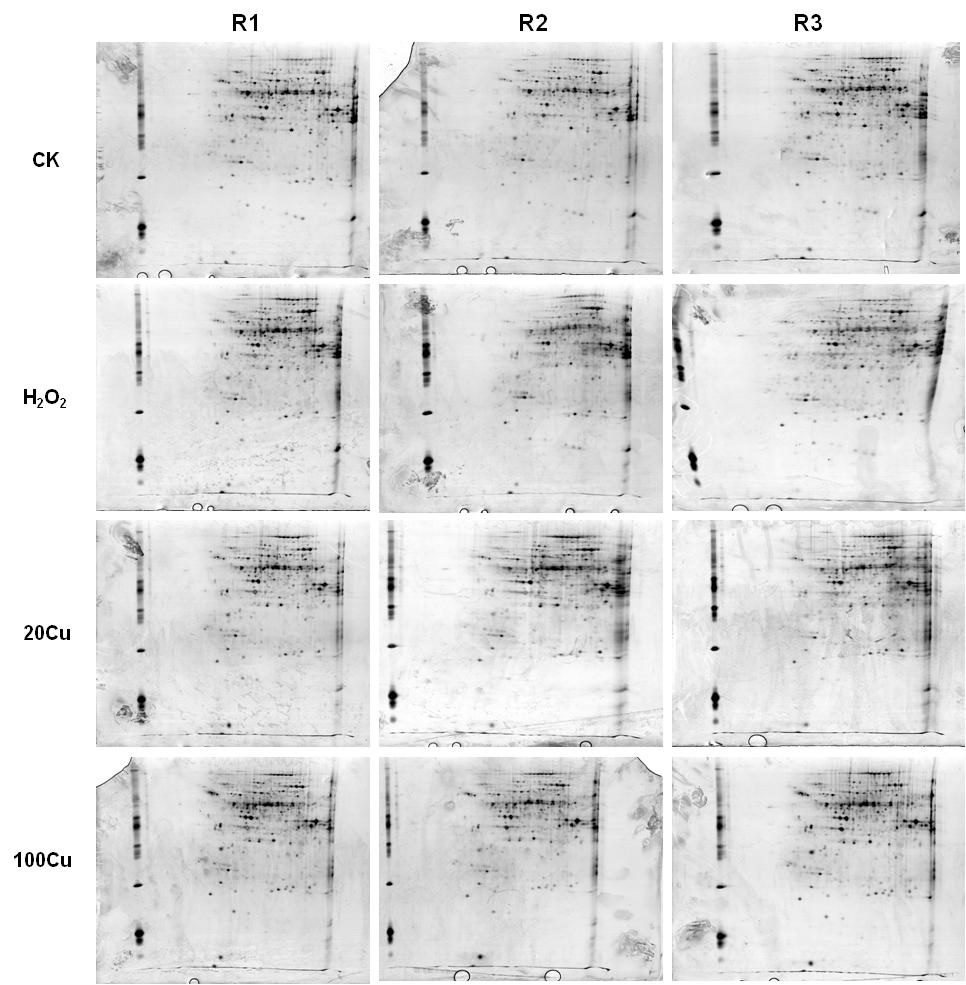

Supplement: Supplementary file 1 [file DataSheet1.zip › Data Sheet-zhang/Supplementary Figure S3.jpg]

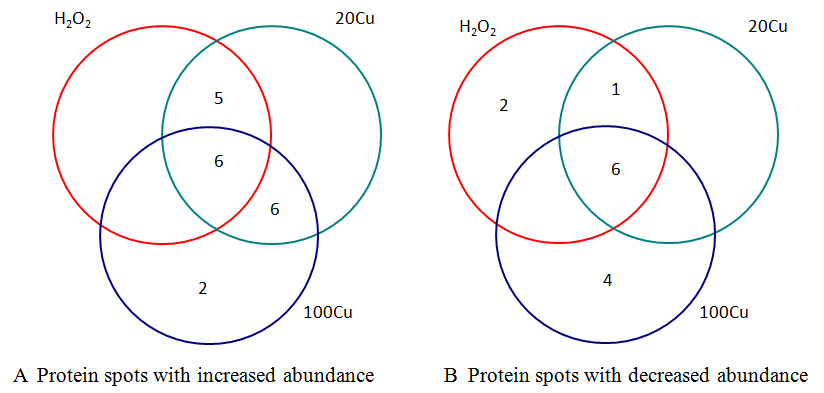

Supplement: Supplementary file 1 [file DataSheet1.zip › Data Sheet-zhang/Supplementary Figure S4.jpg]
